# Multidisciplinary Imaging Review Conference Improves Neuro-oncology Radiation Treatment Planning and Follow-up

**DOI:** 10.7759/cureus.5882

**Published:** 2019-10-10

**Authors:** Dinesh Rao, Peter Fiester, Jeet Patel, Michael Rutenberg, Adam Holtzman, Roi Dagan, Ronny L Rotondo, Sukhwinder Johnny S Sandhu

**Affiliations:** 1 Neuroradiology, University of Florida Health, Jacksonville, USA; 2 Radiation Oncology, University of Florida Health, Jacksonville, USA; 3 Radiation Oncology, The University of Florida Proton Health Therapy Institute, Jacksonville, USA; 4 Radiation Oncology, The University of Florida Health Proton Therapy Institute, Jacksonville, USA; 5 Radiology, Mayo Clinic, Jacksonville, USA

**Keywords:** tumor boards, radiation oncology, head and neck cancer, skull base tumor

## Abstract

Purpose: To review the impact of a weekly multidisciplinary neuroradiology imaging review on the management of patients undergoing radiotherapy.

Methods: A prospective study of the management of 118 patients (30=head and neck, 40=skull base, central nervous system=48) was conducted over a 12-month period from January 2018 through January 2019. After review of each patient’s history and relevant imaging, a radiation oncologist completed a form detailing the changes that were made in diagnosis and management. Imaging source (external and internal examinations), availability of outside reports, report timeliness, the value of reports, changes in interpretation, changes in clinical management, and changes in prognosis were documented. Changes in interpretation and management were designated as major or minor depending on the significance of the change. The managing radiation oncologist indicated whether the imaging review conference substituted for a peer-to-peer consultation with a neuroradiologist.

Results: Nearly half (47%) of all patients had a change in interpretation. Of those, 32% of patients had a major change in interpretation, while 14% had a minor change in interpretation. The existence of the multidisciplinary imaging review conference prevented a peer-to-peer consultation (interruption) by the radiation oncologists to the neuroradiologists in 90% of the cases presented. Further analysis was performed.

Conclusion: The involvement of neuroradiologists in a joint radiation oncology imaging review conference resulted in changes in diagnostic imaging interpretation that led to significant changes in management, expected prognosis, and workflow.

## Introduction

Malignant tumors of the central nervous system (CNS), skull base (SB), and head and neck (HN) represent a wide-ranging spectrum of pathology that may be successfully treated with radiotherapy. Given the need for highly specialized care, patients with disease of these subsites are often treated via a multimodality approach that includes surgery, chemotherapy, and radiotherapy since recent studies have shown that multimodal therapy increases disease-free and long-term survival [1,2]. For some of these pathologies, clinical centers with high case volumes and experience have been demonstrated to provide treatment that results in increased disease-free and overall survival [3].

Multidisciplinary tumor boards (MTB), consisting of surgeons, medical oncologists, radiation oncologists, pathologists, and radiologists, have become more prevalent in recent years and now represent a new standard of care in the treatment of cancer [4-6]. Previous studies of MTB efficacy in the treatment of lung, colorectal, and genitourinary cancer have demonstrated how tumor board meetings can optimize treatment planning [7-9]. Prior studies have also demonstrated the positive impact of MTB in the setting of HN cancer [10,11]. In addition, other studies have examined the effect of reinterpretation of external cross-sectional imaging during multidisciplinary HN tumor boards, demonstrating significant changes to staging, management, and prognosis [12]. HN as well as SB anatomy is particularly complex, and diagnostic imaging interpretation is critical to oncology diagnosis and radiation oncology treatment planning and clinical follow-up. To our knowledge, no studies have isolated the impact of radiology review of cases both from and outside the home institution. Furthermore, the utility of a tumor board from preventing interruptions to neuroradiologist daily workflow has not been studied.

Because imaging is essential for optimal diagnosis, treatment planning, and management, we sought to quantify the impact of a multidisciplinary imaging review conference (MIRC) specifically with regard to imaging review between radiation oncologists and neuroradiologists. 

## Materials and methods

Subjects

We reviewed 118 consecutive patients with SB, CNS, and HN tumors at an MIRC from January 2018 through January 2019 at the University of Florida College of Medicine - Jacksonville as part of an Institutional Review Board (IRB)-approved Practice Quality Improvement (PQI) project. All patients had been referred to one of several radiation oncologists and had either already received, or were planning to receive radiotherapy. All cases were presented at the discretion of the treating radiation oncologist. The sole inclusion criterion for study subject for this project was being presented at the MIRC. Patients were excluded if they had been previously presented for review.

Imaging review conference

The MIRC was established at the University of Florida College of Medicine - Jacksonville in December 2017 with the purpose of reviewing treatment planning and follow-up management issues on a weekly basis. The meeting was borne out of the need for extensive neuroradiology imaging review of highly complex CNS/HN/SB oncology cases for radiotherapy treatment planning and follow-up. Each conference lasted approximately one hour, and a minimum of one radiation oncologist and one fellowship trained neuroradiologist with a certificate of added qualification were present. Typically, three to four specialized radiation oncologists with a focus on CNS/SB or HN oncology were present. Imaging studies for review consisted of diagnostic computed tomography (CT), magnetic resonance imaging (MRI), radiation planning MRI and CT scans with target delineation volumes, and imaging from both external and internal sources. The typical number of patients presented ranged from one to nine, with an average of 3.75 per meeting.

Data collection

This project was initially registered as a PQI study by the IRB at the University of Florida. A questionnaire was developed by both radiation oncology and neuroradiology faculty. Data were de-identified, with the forms filled out in real time by each radiation oncologist. The following data points were recorded: 1. Type of malignancy. 2. Imaging source (external/internal/both). 3. Was a dictated report available?. 4. Did the radiologist’s report answer the clinical question? 5. Was the report timely? 6. Was the outside radiologist contacted? 7. Change in interpretation (major/minor/no change). 8. Change in management (major/minor/no change). Type of change in management (a. RT plan, B. surgery, C. chemo, D. follow-up interval/type, E. avoid further treatment).

Changes in interpretation were designated by the attending radiation oncologist as no change/major change/minor change. A major change was any change in the outside or internal radiologist interpretation that resulted in an overall change in the impression of the imaging examination. For example, if during the review new or residual tumor was present but had previously not been reported. A minor change may represent the extent or dimensions of residual tumor or an incidental finding that required follow-up.

Changes in management were also designated by the attending radiation oncologist as no change/major/minor. Examples of major change included need for or avoidance of more surgery, chemotherapy, or radiotherapy or a change in the radiation treatment plan that would have led to underdosing or undercoverage of the tumor. Minor changes most commonly reflected changes in follow-up or need for additional imaging.

Statistical methods

Frequencies for each of the variables (imaging source, report availability, report answer clinical question, change in interpretation, change in management, change in prognosis, and did the presentation at the MIRC prevent a peer-to-peer consultation with neuroradiology) were calculated. Correlation between imaging source was performed against changes in interpretation, change in management, change in prognosis, report accuracy, prevention of phone call using Fisher’s exact test to calculate p values. Fisher’s exact test was used because of the relatively low sample size. In addition, odds ratio was calculated for statistically significant differences to understand the likelihood of diagnostic accuracy between internal and external imaging reports.

## Results

A total of 118 patients were reviewed. Of those, 105 (90%) patients had malignant tumors and 10% had benign tumors that required evaluation by the radiation oncology service. The most common reasons for presentation at MIRC were to evaluate imaging for radiotherapy planning (n=59), follow-up patients who had been previously treated (n=49) for tumor control versus recurrence, evaluation for tumor progression versus pseudoprogression (n=7), incidental findings (n=2), and infection during treatment (n=1).

Thirty-nine percent of patients had imaging from external facilities. Twenty-two percent of patients had imaging only from our institution. Thirty-eight percent of patients had imaging from both outside institutions and internally. 

**Figure 1:**
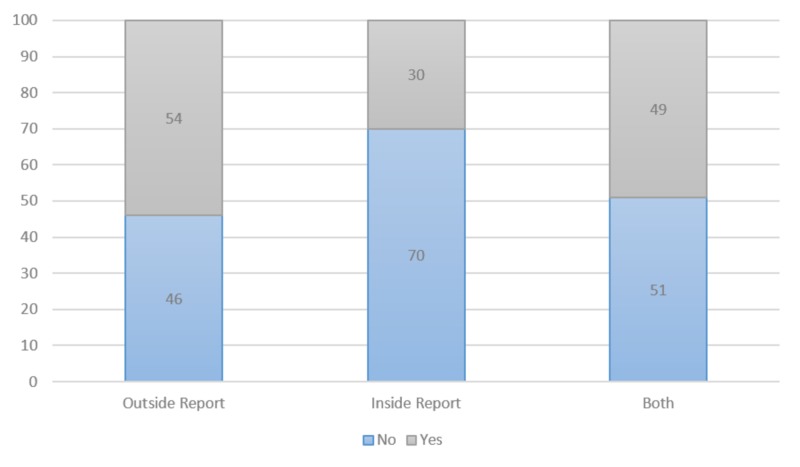
Change in Interpretation (%) 1. Defined as the percentage of cases with any change in the outside or internal radiologist interpretation that resulted in an overall change in the impression of the imaging system. 2. Defined as the extent or dimension of residual tumor or an incidental finding that required follow-up.

Changes in interpretation were taken in aggregate as well as subdivided by imaging source. In aggregate, 32% of patients had a major change in interpretation, 14% had a minor change in interpretation, and 53% had no change in interpretation. Scans performed outside the institution had a higher percentage of changes, with 54% having a major or minor change. Patients with both internal and external sources of imaging had a 49% major or minor change, while 30% of internal scans had a major or minor change. Major and minor changes in interpretation were grouped together for source of imaging report (Figure 1). There was no statistically significant difference between the source of scans and the percentage of change (p<0.11).

**Figure 2:**
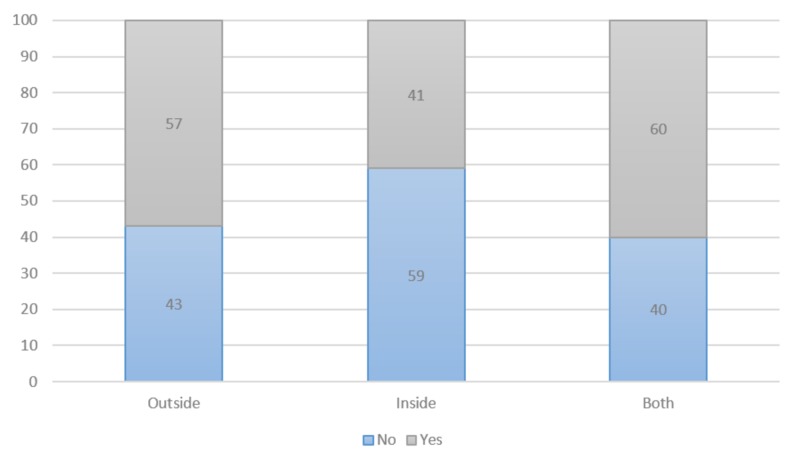
Change in Management (%) 1. Defined the percentage of cases in which a change in the outside or internal radiologist interpretation that resulted in an overall change in the impression of the imaging examination. 2. Defined as need for or avoidance of more surgery, chemotherapy, or radiotherapy or a change in the radiation treatment plan that would have led to underdosing or undercoverage of the tumor.

Changes in management were analyzed the same manner. In aggregate, 40% of patients had a major change in management, 14% had a minor change, and 46% had no change. Scans performed from external sources had major or minor changes in 57% of the cases reviewed, while those performed internally had changes in management 41% of the time. Patients with both internal and external scans had their management change 60% of the time. Major and minor changes in management were grouped together for source of imaging report (Figure 2). There was no statistically significant difference between the source of the scans and the percentage of patients with management change (p<0.27).

Changes in management were correlated against external and internal sources of imaging to determine if the outside interpretation of scans resulted in more management changes versus scans performed internally. There was no significant difference between changes in management based on the source of imaging (p<0.2).

**Figure 3:**
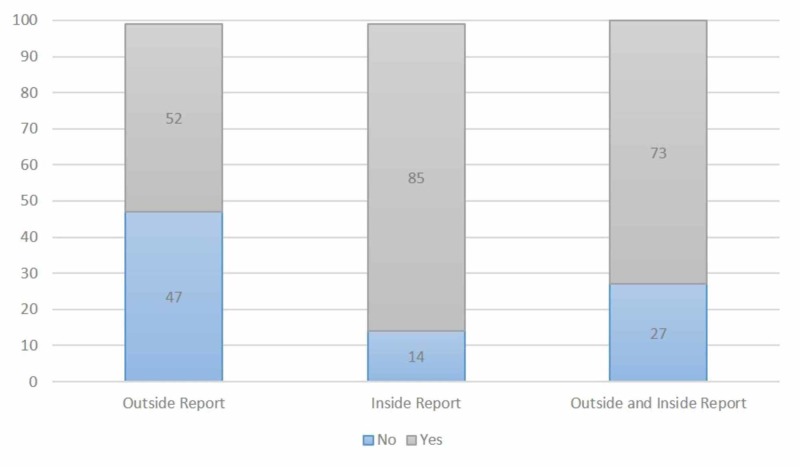
Percentage of Reports that Answers the Clinical Inquiry 1. Radiology reports interpreted by internal radiologists were significantly more likely to answer the relevant clinical question required for patient management (p=0.0089).

The source of imaging was also correlated to whether or not the reports answered the clinical questions the radiation oncologists were seeking (Figure 3). The odds ratio of an inpatient scan being interpreted in a way that answered the clinical question was 5.7 greater than an outside scan (p<0.007; 95% confidence interval, 1.54-17.65).

Thirty-two percent of patients had a change in prognosis as a result of the conference. Approximately half had an improved prognosis (n=19), while the other half had a worsened prognosis (n=19). There was no statistically significant difference between the source of imaging and prognosis (p<0.46).

The radiation oncologists indicated that the existence of the conference prevented a direct peer-to-peer phone call to a neuroradiologist during regular clinical work hours in 90% (n=106) of the cases reviewed. There was no statistically significant difference between the source of imaging and the probability of the radiation oncologist from calling the reading room (p<0.26).

## Discussion

MTB are required for cancer center accreditation [13]. This study aimed to determine the utility of MIRCs in changing management decisions of consulting radiation oncologists. Management of SB and CNS malignancies depends on the accurate interpretation of imaging studies. This often involves a radiation oncologist making the decision to either target coverage or protect critical adjacent neural structures such as optic nerves or brainstem. 

A multidisciplinary approach has been studied previously. Lamb et al. demonstrated that a multidisciplinary team’s decision making changed management in a significant percentage of patients [14]. This change was driven primarily by changes in diagnosis, staging, and the decision of whether or not to use chemotherapy. To our knowledge, only one study has previously studied the interpretation of imaging in the setting of HN cancer [12] The effect of reinterpretation of internal and external imaging by neuroradiologists has not previously reviewed. 

In tertiary referral centers, patients often have imaging from outside institutions in addition to scans performed in house. In addition, the radiation oncology group performs its own CT and MRI scans for radiation planning and these are not formally reviewed by a radiologist. For scans performed at the home institution, the interpreting neuroradiologist would have access to pertinent patient medical and surgical history, physical examination findings, and access to previous imaging, which may not have been available to outside radiologists. Also, new information was frequently presented during the MIRC, which was not available to in-house radiologists who also changed interpretation. In our experience, new clinical information such as the presence or absence of relevant physical examination findings or nuances about the clinical presentation was commonly presented, and after much discussion led to revisions in image interpretation during the conference. Given the reliance of imaging studies on radiation therapy planning and follow-up assessment for HN/SB/CNS neoplasms, the interactions and communication between neuroradiologists and radiation oncologists are critical. Conventional tumor boards and radiology imaging reports in isolation are often inadequate to make detailed radiotherapy plans, especially when dealing with high-dose radiotherapy delivered to adjacent radiosensitive structures (i.e., optic nerves, brainstem, eloquent brain parenchyma, inner ear, etc.). The fine details of imaging analysis can significantly influence radiation target assessment and treatment planning as well as subsequent post-treatment follow-up imaging assessment for treatment response and disease control. We have found that a focused neuroradiology imaging review conference tailored to the needs of CNS/HN/SB radiation oncologists provided a significant impact in the delivery of care for many patients.

Our results demonstrate that radiation oncologists felt scans performed in house were more likely to answer the clinical questions necessary for proper patient management. Although this may reflect a higher level of diagnostic accuracy on the part of internally interpreted scans, this did not translate into fewer internally interpreted scans resulting in a significantly lower change in interpretation or management. 

As there was no statistical difference between changes in interpretation and management between sources of imaging, information that was not previously available and which presented at each imaging review conference likely impacted the reinterpretation in all three types of patients. We may also have lacked the statistical power to demonstrate the full effect of more accurate reporting. It is important to note that a substantial percentage of patient scans had major and minor changes in image interpretation (outside 36%, internal 30%, both 49%) and management (outside 57%, internal 41%, both 60%). 

Changes in disease prognosis also occurred 32% of the time, with roughly half of the patients improved and worsened. Nearly all of the patients with an improved prognosis had original interpretations that indicated the presence of new or worsening tumor (e.g., tumor progression) but were determined by the MIRC to be stable or improved. Similarly, nearly all of the patients whose prognoses were worsened after the imaging review conference had initial interpretations that missed residual tumor or tumor progression. 

An additional important finding regarding a workflow perspective is that the MIRC prevented the radiation oncologist from having to call a neuroradiologist during working hours in 106 instances. That is to say that the MIRC prevented unscheduled workflow interruptions 106 times, or in 90% of the cases. This observation is worth mentioning, since interruptions of radiologists have been shown to decrease diagnostic accuracy and prolong image interpretation time [15,16]

Our findings indicate a significant role and contribution of a focused neuroradiology review conference to the needs of CNS/SB/HN radiation oncology treatment planning and post-treatment follow-up assessment. The specific needs of the radiation oncologists are not often adequately addressed by formal imaging interpretations, and it is via additional communication and collaboration that radiologists are able to improve the delivery of care to this patient population. These findings are in line with the emphasis in contemporary oncology practice to incorporate MTB and integrated multidisciplinary care.

Our study has several limitations. The first is that we only evaluated patients who were presented in an imaging review conference at a highly-specialized tertiary care center that specializes in the treatment of HN, SB, and CNS malignancies. There is no way to assess a control group of patients who did not undergo presentation. Although we expect that the multidisciplinary approach improves decision making, diagnostic accuracy, and treatment, the current data cannot definitively conclude that the imaging review would have led to clinically meaningful differences in treatment outcome. 

Additionally, the study questions asked only focused on a narrow question regarding the utility of neuroradiologists evaluating imaging for radiation oncologists. The results cannot be readily generalized to other types of radiologists or organ systems. 

Given the nature of the conference, the study did not include the opinions of other types of specialists, including pathologists, surgeons, and medical oncologists who might have provided additional clinical input. 

The present study format is also subject to confirmation bias that is inherent to reviewing internal reports, since the neuroradiologists may have come across their own interpretations during the imaging review conference. To minimize this potential bias, four different neuroradiologists attended the imaging review conference on a rotating basis.

## Conclusions

The involvement of neuroradiologists in the setting of radiation oncology multidisciplinary imaging review conference for HN, SB, and CNS malignancies results in changes in imaging interpretation for patients who have had imaging performed both internally and externally; 40% of the patients at our center had clinically significant changes to their management as a result of this conference. In addition, a novel finding of this study was that such conferences provide an organized setting for re-review or re-interpretation of imaging for radiation oncologists, thus preventing interruptions to radiology workflow from peer-to-peer inquiries by radiation oncologists in 90% of the cases reviewed.

## References

[1] Matzinger O, Zouhair A, Mirimanoff RO, Ozsahin M (2009). Radiochemotherapy in locally advanced squamous cell carcinomas of the head and neck. Clin Oncol (R Coll Radiol).

[2] Bonner JA, Harari PM, Giralt J (2006). Radiotherapy plus cetuximab for squamous-cell carcinoma of the head and neck. N Engl J Med.

[3] Koshy M, Sher DJ, Spiotto M (2017). Association between hospital volume and receipt of treatment and survival in patients with glioblastoma. J Neurooncol.

[4] Stalfors J, Bjorholt I, Westin T (2005). A cost analysis of participation via personal attendance versus telemedicine at a head and neck oncology multidisciplinary team meeting. J Telemed Telecare.

[5] Sidhom MA, Poulsen MG (2006). Multidisciplinary care in oncology: medicolegal implications of group decisions. Lancet Oncol.

[6] Leo F, Venissac N, Poudenx M, Otto J, Mouroux J (2007). Multidisciplinary management of lung cancer: how to test its efficacy. J Thorac Oncol.

[7] Sharma A, Sharp DM, Walker LG, Monson JR (2008). Colorectal MDTs: the team's perspective. Colorectal Dis.

[8] Kurpad R, Kim W, Rathmell WK (2011). A multidisciplinary approach to the management of urologic malignancies: does it influence diagnostic and treatment decisions. Urol Oncol.

[9] Wright FC, De Vito C, Langer B, Hunter A (2007). Multidisciplinary cancer conferences: a systematic review and development of practice standards. Eur J Cancer.

[10] Wheless SA, McKinney KA, Zanation AM (2010). A prospective study of the clinical impact of a multidisciplinary head and neck tumor board. Otolaryngol Head Neck Surg.

[11] Nguyen NP, Vos P, Lee H (2008). Impact of tumor board recommendations on treatment outcome for locally advanced head and neck cancer. Oncology.

[12] Loevner LA, Sonners AI, Schulman BJ (2002). Reinterpretation of cross-sectional images in patients with head and neck cancer in the setting of a multidisciplinary cancer center. AJNR Am J Neuroradiol.

[13] Cancer Commission on Cancer (2016). Cancer Program Standards: Ensuring Patient-Centered Care.

[14] Lamb BW, Sevdalis N, Benn J, Vincent C, Green JS (2013). Multidisciplinary cancer team meeting structure and treatment decisions: a prospective correlational study. Ann Surg Oncol.

[15] Balint BJ, Steenburg SD, Lin H, Shen C, Steele JL, Gunderman RB (2014). Do telephone call interruptions have an impact on radiology resident diagnostic accuracy. Acad Radiol.

[16] Wynn RM, Howe JL, Kelahan LC, Fong A, Filice RW, Ratwani RM (2018). The impact of interruptions on chest radiograph interpretation: effects on reading time and accuracy. Acad Radiol.

